# Experimental and Numerical Insights into the Multi-Impact Response of Cork Agglomerates

**DOI:** 10.3390/ma17194772

**Published:** 2024-09-28

**Authors:** Guilherme J. Antunes e Sousa, Afonso J. C. Silva, Gabriel F. Serra, Fábio A. O. Fernandes, Susana P. Silva, Ricardo J. Alves de Sousa

**Affiliations:** 1 Centre for Mechanical Technology and Automation (TEMA), Department of Mechanical Engineering, Campus Universitário de Santiago, University of Aveiro, 3810-193 Aveiro, Portugal; gui.sousa@ua.pt (G.J.A.e.S.); afonso.carvalho.silva@ua.pt (A.J.C.S.); gfserra92@ua.pt (G.F.S.); fabiofernandes@ua.pt (F.A.O.F.); 2 LASI—Intelligent Systems Associate Laboratory, 4800-058 Guimarães, Portugal; 3 Amorim Cork Composites, Rua Comendador Américo Ferreira Amorim, 260, 4535-186 Mozelos, Portugal; susana.silva@amorim.com

**Keywords:** finite element analysis (FEA), cork agglomerates, mechanical behaviour, Mullins effect

## Abstract

Due to their extraordinary qualities, including fire resistance, excellent crashworthiness, low thermal conductivity, permeability, non-toxicity, and reduced density, cellular materials have found extensive use in various engineering applications. This study uses a finite element analysis (FEA) to model the dynamic compressive behaviour of agglomerated cork to ascertain how its material density and stress relaxation behaviour are related. Adding the Mullins effect into the constitutive modelling of impact tests, its rebound phase and subsequent second impact were further examined and simulated. Quasi-static and dynamic compression tests were used to evaluate the mechanical properties of three distinct agglomerated cork composite samples to feed the numerical model. According to the results, agglomerated cork has a significant capacity for elastic rebound, especially under dynamic strain rates, with minimal permanent deformation. For instance, the minimum value of its bounce-back energy is 11.8% of the initial kinetic energy, and its maximum permanent plastic deformation is less than 10%. The material’s model simulation adequately depicts the agglomerated cork’s response to initial and follow-up impacts by accurately reproducing the material’s dynamic compressive behaviour. In terms of innovation, this work stands out since it tackles the rebounding phenomena, which was not previously investigated in this group’s prior publication, either numerically or experimentally. Thus, this group has expanded the research on cork materials’ attributes.

## 1. Introduction

Cellular materials have been successfully employed in various fields, including building and aerospace. These materials have mainly been used in engineering applications where a high ratio between mechanical properties and weight is desirable. Their essential features include qualities like damping, insulation, and crashworthiness, to name a few [1,2].

Cellular materials are divided various subcategories, including natural, synthetic, open-cell, and closed-cell materials [3]. In terms of the internal porous nature of these materials, their cellular pores may be open or closed. Open pores share only their edges, whereas closed pores share edges and cell walls. They share the ability to absorb significant energy by deforming under compressive loads while maintaining low stress levels. Their uniaxial compressive stress–strain curves typically show a linear elastic zone, a plateau region, and a densification zone. These materials are commonly employed in protective and packaging applications (e.g., motorcycle helmets [4]). Cellular materials include expanded polypropylene (EPP), polystyrene (EPS), and metal foams. The choice of material depends on its specific application, as factors like density, strain rate, and chemical composition can significantly influence its mechanical properties. Researchers often study these materials under quasi-static and dynamic stress due to their practical significance [5,6]. Synthetic cellular materials excel in single-impact applications, absorbing more energy and reducing acceleration peaks [7]. However, some synthetic materials, like EPS, offer limited protection in cases of a second impact, as they deform mainly plastically and exhibit minimal elastic recovery [8].

Recent awareness of going green and demands regarding more sustainable production patterns are promoting the usage of natural alternatives to synthetic cellular materials. In this regard, cork, a naturally occurring cellular substance with significant crashworthiness and insulation properties, is a material that is the natural choice to replace synthetic materials. Typically, its cells consist of closed hexagonal prisms arranged in rows so that two cells share the same hexagonal face. Nevertheless, the rows are spaced apart such that the membranes covering the hexagonal faces are not continuous throughout the rows. Cork, in its agglomerated form, is distinguished by having both a high viscoelastic return and a good energy absorption capacity, which indicates that after an impact, this material’s ability to continue absorbing energy is essentially intact and it deforms more elastically [9,10]. In addition to its excellent compressibility and dimensional recovery, cork exhibits strong chemical stability, excellent insulating qualities, and a very low permeability to liquids and gasses [11]. Strong interest has been raised in using agglomerated cork to replace synthetic cellular materials because of its sustainability, low carbon footprint, and alignment with the Sustainable Development Goals (SDGs).

Researchers looking for new uses for this eco-friendly, sustainable material have been active [12,13]. Researchers [14] have explored the use of agglomerated cork stoppers for still wines and spirits in the packaging sector. A non-supervised exploratory analysis was conducted on various cork samples, revealing that only 4 or 5 initial parameters are needed to determine the appropriate stopper. This suggests that fewer criteria are needed to accurately characterize the mechanical properties of agglomerated cork, enabling the selection of the best material for the intended use. Serra et al. [15] created a multi-layer helmet based on agglomerated cork that provides energy absorption and protection levels comparable to current helmets on the market, but with a substantially lower environmental impact. According to research, cork can absorb energy similarly to EPS and withstand multiple impacts because of its ability to withstand a large amount of deformation without causing significant harm to its viscoelastic cellular structure. This allows the cork to recover after loading [16]. In addition, multiple models with varying features were produced, and impact testing was carried out in an accredited laboratory. This helmet addresses the absence of material recovery or recycling and signals a change toward sustainability in an industry that relies heavily on petroleum-based components. Compared to regular bike helmets, the materials used, the ease of dismantling, and its recycling produce 42% fewer carbon emissions. Another investigative team [17] developed novel homogenous composite materials based on agglomerated cork infused with non-Newtonian fluids (shear thickening) to prevent cell crushing and boost energy absorption. The researchers discovered that samples containing 10 wt. % could bear loads from both a first and second hit without exhibiting macroscopic fissuring or any other apparent effects. This demonstrates that it is possible, by maintaining their fluid and structural integrity, to create samples that can endure many hits, significantly reducing user damage, and that are far more sustainable than those currently employed in body safety applications.

Natural cork is a complex biological substance [11]. Numerous studies have assessed the essential characteristics of cork’s mechanical behaviour, such as Young’s modulus, Poisson’s ratio, plateau stress, densification strain, and energy density, under quasi-static axial compressive loads [18,19] and under dynamic circumstances [20]. Anjos et al. [21] have researched the impact of cork density on the material’s mechanical behaviour, such as its Young’s modulus, stress plateau, and the shape of the stress–strain curve, both when compressed and after the subsequent recovery of its dimensions. The investigative team found out that the density influenced the compression such that the corks with a high density presented higher stiffness, but the dimension recovery was higher for corks with a low density. Santos et al. [22] investigated the effects of density, resin type, binder weight percentage, and grain size on the mechanical properties of composite materials based on agglomerated cork under compression and impact. The study team came to the following conclusions: larger grains produce composites with a higher plateau stress and Young’s modulus; increased density leads to a higher plateau stress but a lower densification deformation; and the mass proportion of the binder delays the densification stage. However, only a few researchers examined the mechanical behaviour of cork under dynamic compressions. Gameiro et al. [23] investigated the mechanical behaviour of cork (as a filler for square aluminium tubes), specifically in terms of its Young’s modulus, stress plateau, and energy density (natural and agglomerated), under impact loading at strain rates ranging from 200 to 600/s. The scientists noted that fillers like agglomerate and micro-agglomerate cork provided clear benefits, in terms of energy absorption and deceleration, for the square aluminium tubes under study. Additionally, cork is less expensive and lighter than some metallic foams, which may encourage the usage of cork in innovative lightweight energy-absorbent structures. Its recovery dimensions at dynamic rates, however, were not investigated. Cork’s compressive behaviour under impact was simulated by Fernandes et al. [24], along with the material’s relaxation following dynamic compression, using both the Hyperfoam material model and the Mullins effect material model available in Abaqus. A finite element analysis (FEA) was used for the numerical simulations, and the resultant material model was evaluated against the outcomes of the experiments. As a preliminary validation, the authors numerically reproduced the experiments performed in [23] via an FEA and obtained good accuracy. After validation, a dynamic test resorting to a drop tower was carried out successfully, validating the model and adequately representing cork’s mechanical behaviour under dynamic compressions. The Hyperfoam and Mullins effect material models worked together to replicate the actual behaviour of agglomerated cork during compression and the relaxation that followed.

Several academics have concentrated on creating numerical models that mirror the mechanical behaviour of cork agglomerates in various situations. Gomez et al. [25] investigated the behaviour of sandwich panels made of cork cores and carbon/epoxy face sheets when impacted at intermediate velocities. The researchers used a nonlinear/explicit finite element model and applied continuous damage models to predict how damage occurs within and between the layers of face sheets. Additionally, they employed a hyperelastic elastomeric foam model with multiaxial failure criteria to describe the fundamental material behaviour of the panels. The progression of the face sheet’s intra- and inter-laminar damage was predicted using continuous damage models. Using a continuum damage model (CDM) approach for fabric-reinforced composites, which was implemented in Abaqus/explicit through the built-in VUMAT subroutine ABQ_PLY, developed by Jhonson [26] based on the Ladeveze et Le-Dantec damage continuum model [27], the intra-laminar damage in the face sheets was modelled. Ply is an orthotropic elastic material that can withstand plastic deformation under shear force and gradual deterioration due to fibre/matrix cracking. The Tsai–Wu fracture criteria were used with a VUSDFLD user function to implement element deletion [28]. This numerical model successfully replicated the actual behaviour of the ply. Another research team led by Sergi et al. [29] investigated the impact response to puncture of bio-based sandwich structures with an intraply flax/basalt hybrid core and agglomerated cork core. The excellent agreement between an FEA and the experimental results ensures a trustworthy prediction of the dynamic response of the core. The same researcher [30] also performed a finite element analysis on the high-velocity impact responses of sandwich materials made with PVC foam and agglomerated cork. Alcântara et al. [1] performed a constitutive and numerical study to evaluate the energy absorption capabilities of metallic tube structures that have an agglomerated cork core to uncover new applications for this composite material.

Despite the intensive work in this area, no computational model can simulate the mechanical response of composite materials made of cork agglomerates when subjected to repetitive loads. This work intends to fill this knowledge gap. While superb numerically, Fernandes et al.’s work [24] can only simulate a single impact. This means that the dimensional recovery potential of agglomerated cork composites, which can withstand multiple impacts with essentially no plastic deformation, is not implemented. This is important since cork-based composite materials, due to their ability to absorb energy after various impacts, have the possibility of replacing, in an environmentally sustainable way, the current synthetic materials used in safety applications, such as EPS, which deforms permanently after its first impact and must be replaced immediately.

In this study, the dynamic compressive behaviour of agglomerated cork, and specifically its Young’s modulus, stress plateau, stress–strain curve, and energy density, was simulated using a finite element analysis (FEA), as was the relaxation of the material during its unloading. It also determined the relationship between the sample’s density, relaxation behaviour, and Mullins effect material parameters, with the type of binder, weight percentage of binder, and grain size being independent parameters. Additionally, as opposed to Fernandes’s numerical model [24], the simulation was run while considering gravity.

## 2. Materials and Methods

**Figure 1:**
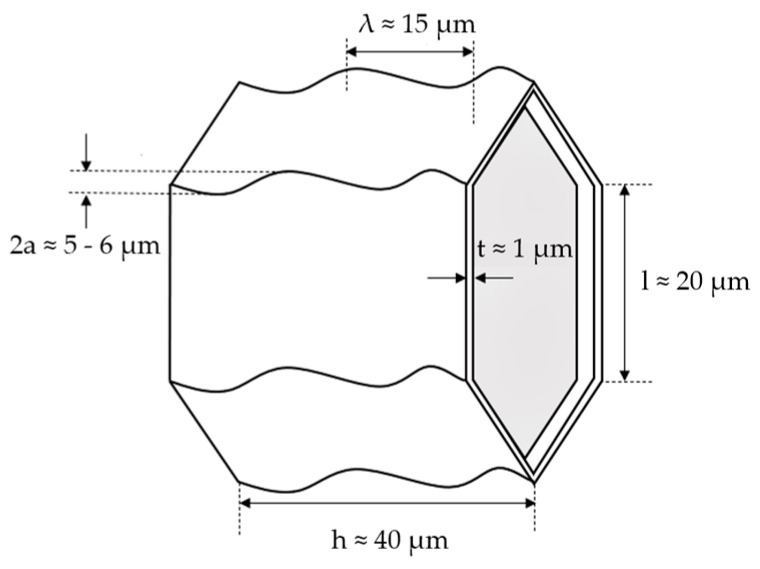
Ideal cork cell topology and size parameters.

Cork can be categorized as a honeycomb substance made of approximately hexagonal, prismatic, closed cells arranged to produce rows parallel to the tree’s radial growth direction. Approximately 15% of the overall volume of the material is made up of these closed compartments [24]. As can be seen in Figure 1, the cell walls exhibit substantial corrugations along the axis of the prism. These undulations and corrugations provide the cell walls with considerable flexibility, which dictates the mechanical behaviour of the cork [11].

Agglomerated cork, a byproduct of cork stopper production, is employed in this investigation. It can be produced in moulded blocks, and parameters such as its density, grain size, and binder can be tuned to achieve desirable properties [22]. Due to the random orientation of its grains, and its small grains (0.5–2.0 mm), its mechanical and thermal behaviour is practically isotropic, which is an advantage compared to the anisotropic behaviour of natural cork. Furthermore, natural cork has a limited range of dimensions because of its extraction process.

### 2.1. Fundamentals and Constitutive Laws

NOTE: The Abaqus Analysis User’s Manual [31] is the source of all equations that are shown in this section.

Abaqus (Dassault Systèmes Simulia Corp., Vélizy-Villacoublay, France) provides a material library suitable for modelling a wide range of materials [31]. Agglomerated cork, under compression, mainly displays a visco-hyperelastic behaviour, with plastic behaviour making up a relatively minor portion of the material’s mechanical properties and only occurring at very high deformations and strain energies. Modelling agglomerated cork with Abaqus (version 2017) nonlinear hyperelastic models is a solid starting point. In doing so, the Hyperfoam material model was employed. This is a typical isotropic and nonlinear model used to describe the hyperelastic behaviour of elastomeric foams. It is also designed for applications involving finite strains of up to 90% during uniaxial compression.

The isotropic elastic properties of a hyperelastic material model may be expressed in terms of a strain energy (stored energy) function, which is generally a function of the three invariants of each of the two Cauchy–Green deformation tensors and given in terms of their primary extension ratios, or stretches. Due to their distinctive characteristics and properties, several strain energy formulations are suitable for describing various hyperelastic material systems [32]. The elastic behaviour of the foams in the Hyperfoam material model relies upon the following strain energy function:

(1) $$\tilde{\;U_{s}}=\sum_{i=1}^{N}\frac{2\mu_{i}}{\alpha_{i}^{2}}\left[\lambda_{1}^{\alpha_{i}}+\lambda_{2}^{\alpha_{i}}+\lambda_{3}^{\alpha_{i}}-3+\frac{1}{\beta_{i}}\left({\left(J\right)}^{{-\alpha }_{i}\beta_{i}}-1\right)\right]\;$$

where *N* is a material parameter related to the polynomial order, *λ_i_* are the principal stretches, and *J* is the elastic volume ratio, given by

(2) $$J=\lambda_{1}\lambda_{2}\lambda_{3}$$

The shear modulus is given by *µ_i_*, whereas *α_i_* and *β_i_* are curve-fitting non-integral exponents. The initial shear modulus, *µ*_0_, and the coefficients *µ_i_* are linked by

(3) $$\mu_{0}=\sum_{i=1}^{N}\mu_{i}\;$$

The last-mentioned symbols, *β_i_*, are concerned with the compressibility of the material, and the initial bulk modulus, *K*_0_, is determined by the following expression:

(4) $$K_{0}=\sum_{i=1}^{N}2\mu_{i}\left(\frac{1}{3}+\beta_{i}\right)\;$$

The coefficients *β_i_* define how compressible each term in the energy function is. The following expressions connect *β_i_* and *ν_i_*, which is Poisson’s ratio:

(5) $$\beta_{i}=\frac{v_{i}}{1-2v_{i}}$$

(6) $$\nu_{i}=\frac{\beta_{i}}{1-2\beta_{i}}\;$$

So, if *β_i_* is constant over all terms, there is only one effective Poisson’s ratio, *ν*. This effective Poisson ratio holds true for finite values of the principal logarithmic strains *ε*_1_, *ε*_2_, and *ε*_3_. Under uniaxial tension, the equation (*ε*_2_ = *ε*_3_ = −*ν* *ε*_1_) is valid. The primary nominal strains are correlated to the principal stretches by the following expression:

(7) $$\varepsilon_{i}=\lambda_{i}-1\;$$

The Mullins effect model is used with the Hyperfoam material model to extend the isotropic elastomeric foam model and accurately represent the long-term energy dissipation and stress softening effects in agglomerated cork. The Mullins effect material model that Abaqus offers is designed to simulate the phenomenon of stress softening, which is frequently seen in filled rubber elastomers due to strain-related degradation. The stress required on reloading is less than that of the first loading for stretches up to the maximum stretch achieved on the initial loading when an elastomeric test specimen is exposed to simple tension from its virgin state, unloaded, and reloaded. The Mullins effect is a phenomenon that reduces stress.

**Figure 2:**
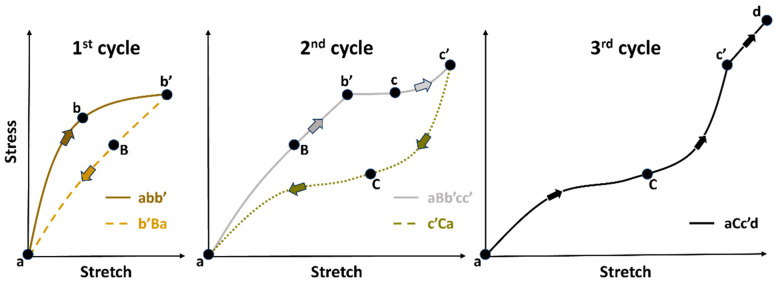
A typical elastomeric foam’s stress–stretch response with energy dissipation—adapted from [33].

Figure 2 is based on research by Ogden and Roxburgh [33]. Consider the main loading path (abb’) of a previously unstressed material with loading up to point (b’). Unloading from b’ follows the path b’Ba. The softened path is retraced as aBb’ when the material is loaded again. For further loads, this procedure is repeated (abb’ → b’Ba → aBb’cc’ → c’Ca → aCc’d). This is a prime example of the Mullins effect. The loading route abb’cc’d shall hereafter be known as the “primary material response,” and the constitutive behaviour of the primary response can be specified using the standard energy potentials of the hyperelasticity models in Abaqus [33].

This material model simulates the energy absorption in foam components subjected to dynamic loading at rapid deformation rates relative to their typical foam relaxation time. In these circumstances, it is reasonable to presume that the foam material has sustained long-term damage. An enhanced strain energy density function with the following formula is introduced into this model to account for the impacts of energy dissipation:

(8) $$U_{s}\left(\lambda_{i},\eta \right)=\eta \tilde{U_{s}}\left(\lambda_{i}\right)+\phi \left(\eta \right)$$

where *λ_i_* (*i* = 1, 2, 3) symbolizes the main mechanical stretches and *Ũ_s_* (*λ*_*i*_) is the strain energy potential of the essential foam behaviour defined by Equation $\left(1\right)$. The function *ϕ*(*η*) is a continuous function of the damage variable, *η*, and is referred to as the “damage function.” Throughout the deformation, the damage variable, *η*, fluctuates constantly within 0 < *η* < 1. The damage function *ϕ*(*η*) must become *ϕ*(*η*) = 0 when the damage variable is equal to one, which makes the material’s deformation state reliant on the curve that depicts its primary foam behaviour *U_s_* (*λ_i_*, 1) = *Ũ_s_* (*λ_i_*). Hence, the augmented energy function then reduces to the strain energy potential of the primary foam behaviour, and only the Hyperfoam material model is, therefore, capable of simulating the material’s mechanical behaviour.

Considering the Mullins effect, the stresses can be calculated by

(9) $$\sigma \left(\lambda_{i},\eta \right)=\eta \tilde{\sigma }\left(\lambda_{i}\right)\;$$

where $\tilde{\sigma }$ is the stress corresponding to the principal foam behaviour at the current deformation level *λ_i_*; therefore, the stress is calculated by simply multiplying the damage variable, *η*, by the stress of the essential foam behaviour. The model predicts unloading and reloading from any given strain level along a single curve that passes through the stress–strain plot’s origin and generally deviates from the material’s principal behaviour. Additionally, the model forecasts energy loss in the case of volumetric deformation only.

With respect to the deformation, the damage variable, *η*, varies as follows:

(10) $$\eta =1-\frac{1}{r}erf\left(\frac{U_{s}^{m}-U_{s}}{m+\beta U_{s}^{m}}\right)\;$$

where *r*, *β*, and *m* are material parameters, $U_{s}^{m}$ is the greatest value of *Ũ_s_* at a specific moment in a material’s deformation history, and *erf(x)* is the error function. In contrast to the dimensionless parameters *r* and *β*, the parameter *m* has stress-related dimensions. The constraints *r* > 1, *β* ≥ 0, and *m* ≥ 0 (the parameters *β* and *m* cannot both be zero) apply to these material parameters. 

Generally speaking, there are no apparent physical meanings for these metrics. The *m* parameter describes the rate of material deterioration following unloading. However, a nonzero *m* leads to little or no damage at low strain levels. The parameter *r* controls the amount of damage; the larger the *r* value, the less the damage is. The *β* parameter decreases the stress reduction following a strain reversal. 

**Figure 3:**
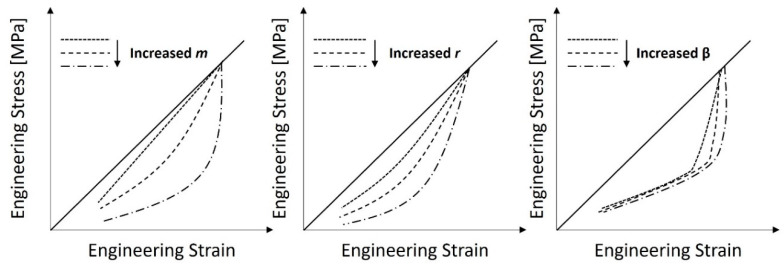
The influence of Mullins effect material parameters on the relaxation stage—adapted from [34].

The PolymerFEM [34] website offers a numerical parametric analysis of the Mullins effect model. The effects of the three parameters (*r*, *m,* and *β*) of the Ogden–Roxburgh–Mullins Damage model on the projected stress–strain response were investigated and are graphically displayed below. Figure 3, while simply suggestive, helps to explain our conclusions.

When *η* = 1 is attained, then *Ũ_s_* = $U_{s}^{m}$, corresponding to a point on the primary curve. Conversely, the damage variable, *η*, achieves its minimum value, *η_m_*, when deformation is eliminated and *Ũ_s_* = 0, as given by

(11) $$\eta_{m}=1-\frac{1}{r}erf\left(\frac{U_{s}^{m}}{m+\beta U_{s}^{m}}\right)\;$$

The value of *η* varies repeatedly between 1.0 and *η_m_* for all intermediate values of *Ũ_s_*. By deducting the dissipated energy from the enhanced energy, the recoverable portion of the energy is obtained as follows:

(12) $$U_{recoverable}=\eta \tilde{U_{s}}\left(\lambda_{i}\right)+\phi \left(\eta \right)-\phi \left(\eta_{m}\right)$$

where the energy lost as a result of material damage after the load has been completely unloaded is represented by the residual value of the augmented energy function, *ϕ*(*η*_*m*_).

### 2.2. Finite Element Simulation

Uniaxial compression testing data were described in order to define the strain energy function parameters. Abaqus uses a least squares fitting methodology to calculate the Hyperfoam material parameters. The experimental compression tests carried out at almost static rates were multiplied by a scaling factor (the function of the strain rate), yielding the stress–strain curve imported into Abaqus. This scaling up is conceivable in agglomerated cork since the stress–strain curve for this material has a constant shape [23]. The scale factor employed has a value of 2.1, which is marginally more than the scale factor of value 3 utilized in the simulations of Gameiro et al. [23], with a different FE code (LSDYNA), and the scale factor value of 3.1 employed by Fernandes et al. [24]. Gameiro et al. [22] recommend using a scale factor value of 3 for micro-agglomerated cork (grain sizes of 0.5 to 1.0 mm) and a scale factor value of 2 for standard agglomerated cork (grain sizes of 2.0 to 4.0). A null Poisson coefficient was considered because the experimental testing allows for the conclusion that there is essentially little lateral deformation and, as a result, that it is negligible.

**Figure 4:**
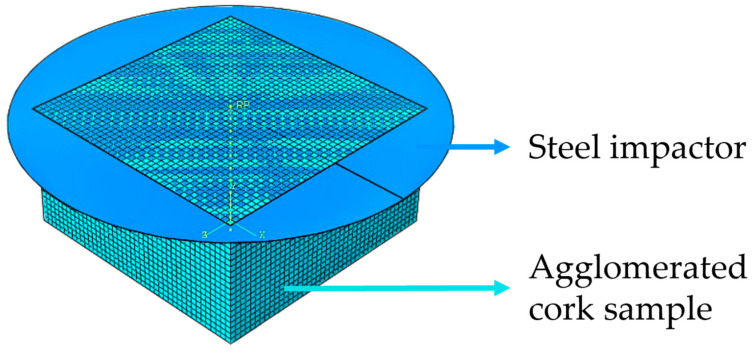
Numerical setup of the drop test.

To numerally simulate the guided drop tests, a sample 50 mm in length, 50 mm in width, and 25 mm in thickness was modelled with the use of reduced integrated, hourglass-stabilized, 8-node hexahedral finite elements (C3D8R). The sample mesh was carefully built to avoid warped and distorted elements to produce accurate results unaffected by mesh element size while still having an appropriate computational processing time. Numerous simulations were run, each time adding more elements until the results converged, to establish the appropriate number of elements for the sample’s mesh. The optimum mesh contained 37,044 elements in total, with 42 elements along each edge’s length and width and 21 elements along each edge’s thickness. Additionally, a model of an analytical rigid body in a disc was modelled to represent the stainless steel impactor. Figure 4 displays this setup. The simulations were carried out using an explicit solution method. No mass scaling was employed during the simulations.

In these experiments, the rigid impactor only had one degree of freedom in the compression direction. A “hard” surface-to-surface contact was used to describe the interaction between the sample and the rigid bodies [24]. The impactor was assigned a predetermined field velocity of 3.84 m/s.

### 2.3. Agglomerated Cork Samples

Amorim Cork Composites (ACC, Mozelos, Portugal) provided the materials for this experimental campaign. The three types of materials made accessible by the ACC will now be referred to as types A, B, and C for confidentiality reasons. The most pertinent details and traits of each type of material are displayed in Table 1. All the samples had a 6–8% moisture content, and all the experiments were conducted in 70–80% relative humidity.

These three distinct types of materials are all composites based on agglomerated cork, with the grain size of types A and C ranging from 2 to 4 mm and that of type B ranging from 2 to 5 mm, whose binder is a hard matrix resin with a polyurethane-based adhesive. In terms of density, type A material weighs 210 kg/m^3^, type B weighs 300 kg/m^3^, and type C weighs 400 kg/m^3^. The percentage of binder by mass is 15 wt.% for type A, 14 wt.% for type B, and 12 wt.% for type C. The same moulding process was used to create all three types of materials. Therefore, the density of the composite is the only factor that can significantly affect the various mechanical behaviours of these materials.

**Figure 5:**
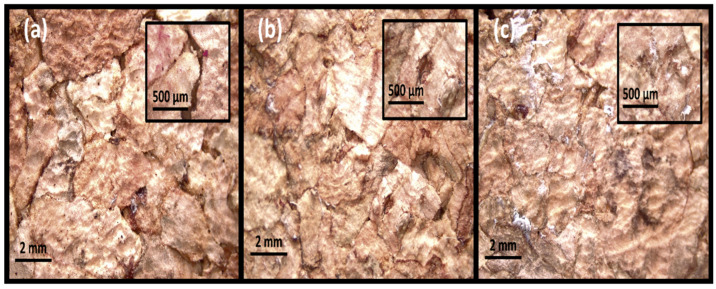
Sample types (before testing) under a microscope at both 8× and 35× magnification: (**a**) type A, (**b**) type B, and (**c**) type C.

The samples were square-faced parallelepipeds measuring 50 mm on one side and 25 mm thick and used for both the impact and quasi-static compression tests. Because the materials from ACC were provided in plates with a 25 mm thickness, a square of 50 × 50 mm guarantees that their mechanical properties are consistent. The three different sample types are depicted in Figure 5 using microscopic amplification at 8× and 35× (in the smallest rectangle). It is easy to see how the samples’ compaction levels differ; sample C has nearly no voids, indicating a higher density and, hence, a greater degree of compaction, while sample A exhibits some voids due to its lower density.

## 3. Experimental Campaign

### 3.1. Uniaxial Quasi-Static Compression Test

**Figure 6:**
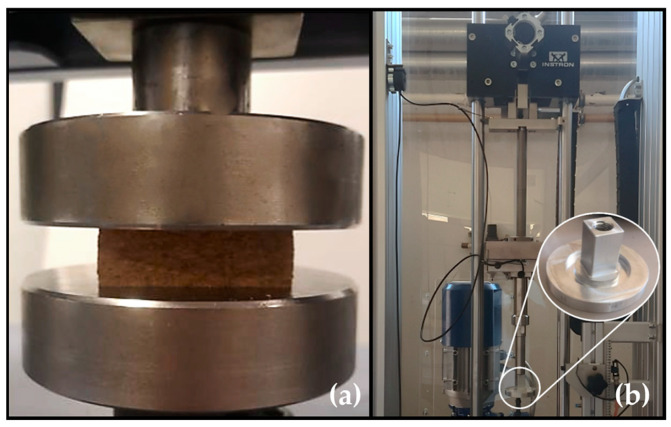
(**a**) Uniaxial quasi-static compression test using a Shimadzu AG-100 kN. (**b**) Instron drop tower and flat impactor.

Uniaxial quasi-static compressive tests were carried out using a Shimadzu AG- 100 kN testing machine, as seen in Figure 6a. The uniaxial compression test lasted until the agglomerated cork densification stage, which is roughly 80% of its nominal strain, or 20 mm of displacement. However, on occasion, it was not possible to attain an 80% nominal extension because of the rapidly increasing force in the densification zone, and the test had to be manually terminated for safety reasons. To guarantee the repeatability of the results, a minimum of four samples of each type of material were evaluated. These samples were precisely centred, and a meagre, continuous strain rate of 3.33 × 10^−3^/s was used to compress them. Two research discoveries led to the adoption of this strain rate. The compressive behaviour of agglomerated cork is nearly strain-rate-independent across strain rates ranging from 9.62 × 10^−4^/s to 4.82 × 10^−3^/s, according to Fernandes et al. [24]. Moreover, Gameiro et al. [23] suggest that when compressed at strain rates between 1.25 × 10^−3^/s and 1.60 × 10^−3^/s, agglomerated cork exhibits strain rate independence. The output force–displacement curves made it possible to calculate the stress–strain curves and the amount of energy absorbed per volume.

### 3.2. Impact Tests

The Instron 9440 (Instron, Pianezza, Italy) drop tower was employed for the impact tests. Two setups were prepared to carry out tests at 60 J and 120 J. The 8.15 kg impactor was raised to a height of around 750 mm for experiments with 60 J impacts. For experiments with 120 J of energy, an additional 5 kg of mass was added, bringing the total mass to 13.15 kg. The impactor was also elevated to a height of roughly 931 mm. It should be noted that the 120 J energy level test was carried out to verify a hypothesis that will be discussed later in the current research.

The impact test drop tower and selected impactor are depicted in Figure 6b. The load, position, and time data were gathered using a 45 kN load cell and a position encoder that were integrated into the machine in order to determine the time–acceleration history, forced displacement, and nominal stress–strain curves of all the samples. Bluehill Impact software (version number 4.26.0.16174) was used to synchronize and process the data. Both a smoothing with ten smoothing points and a CFC1000 digital filter following SAE J211:95 were used to reduce the noise.

The samples were 50 × 50 × 25 mm in size. Following the initial impact, a second impact of the same energy was made with a delay of 30–40 s to allow the impactor to reach its full height. According to Fernandes et al. [24], cork agglomerates’ form recovery happens in milliseconds. Hence, 30–40 s is more than adequate time for the samples to almost completely recover their dimensions. Similar to quasi-static studies, these aim to ascertain how the density of the composites affects the mechanical response of cork agglomerates under dynamic loading.

In order to determine whether a material can withstand several hits, how its mechanical response degrades based on the damage sustained between the first and second impact will also be evaluated. The assessment of energy densities, acceleration peaks, and stress–strain curves will be covered in the section that follows. The energy density (*E_v_)*, or the amount of energy absorbed per unit volume, can be determined by integrating the equation of the stress–strain curve, since the absorbed energy is equal to the area under the stress–strain curve.

## 4. Results and Discussion

### 4.1. Uniaxial Quasi-Static Compressive Tests

**Figure 7:**
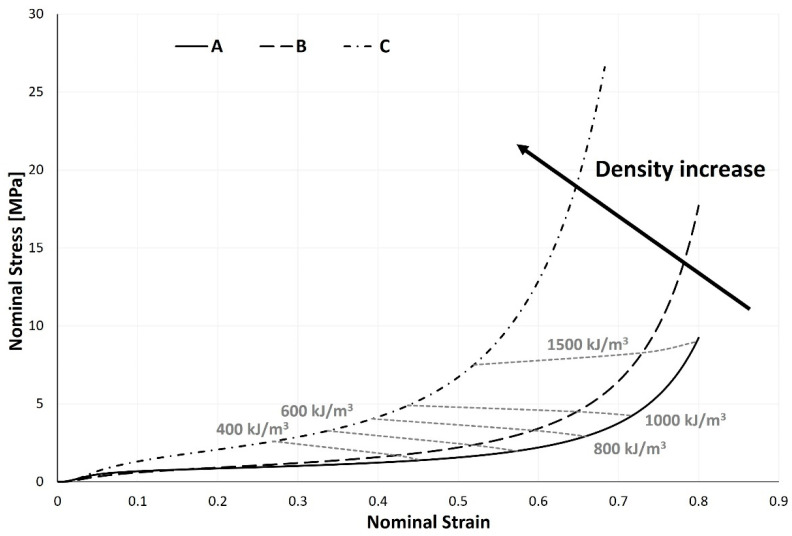
Nominal stress–strain curves of the three samples at five different energy levels.

Beginning this study with the elastic linear zone, an increase in the composite’s density causes an increase in its Young’s modulus, meaning that the sample’s resistance to elastic deformation rises as its density does. Additionally, it is possible to confirm that this zone is reasonably reduced (Figure 7) for samples with lower densities. It is interesting to note that the plateau region flattens out and lengthens as the density of the composite lowers. The densification strain, which marks the transition between the plateau and densification regions, is around 0.5 for a sample with a density of 400 kg/m^3^. In contrast, it is 0.7 for a sample with a density of 210 kg/m^3^. For some applications where longer and less abrupt energy absorption is sought, more extended plateau zones are attractive. As will be seen later, the plateau stress does, however, fall with decreasing density, which results in decreased energy density. In the examination of Figure 7’s last zone, the densification zone, it is conceivable to say that an increase in sample density causes the anticipation of cell crushing. In other words, the lower their composite density, the greater the deformation the samples experience. These findings and conclusions are consistent with the research conducted by Santos et al. [22], where these researchers assessed the impact of their samples’ density on the mechanical behaviour of cork agglomerates under compression, in addition to other factors. This current research extends and supports the findings of Santos et al. [22] because those researchers only looked at densities in a narrow range, between 120 and 200 kg/m^3^.

For the various samples, four absorbed energy density values—400, 600, 800, and 1000 kJ/m^3^—were computed. The energy released during unloading is not factored into the absorbed energy. Given the nature of the isocurves, it is acceptable to approximate them to straight lines. All four isocurves have a negative slope, but as the amount of absorbed energy rises, the slope tends to become smaller. A negative slope shows that, as the sample’s density declines, lower stresses are needed, but more significant deformations are required, to attain the desired energy values. As a demonstration, the isocurve already has a positive slope at an energy density of 1500 kJ/m^3^, indicating that the inflexion point lies between the energy levels of 1000 and 1500 kJ/m^3^. Although they might be challenging to interpret, energy density isocurves are crucial tools in the sample selection process. For some applications and intended energy levels, a lower stress is preferred, at the cost of deformation. However, for other applications, the desired outcome may be the opposite. This is why choosing the optimal sample is tied to the intended application.

However, possibly the most significant finding from examining these isocurves is that larger sample densities result in higher energy densities, as seen in Table 2. Drawing a tangent line to the stress–strain curve in the densification zone and intersecting it with the abscissa axis allowed for the calculation of the densification strain.

**Figure 8:**
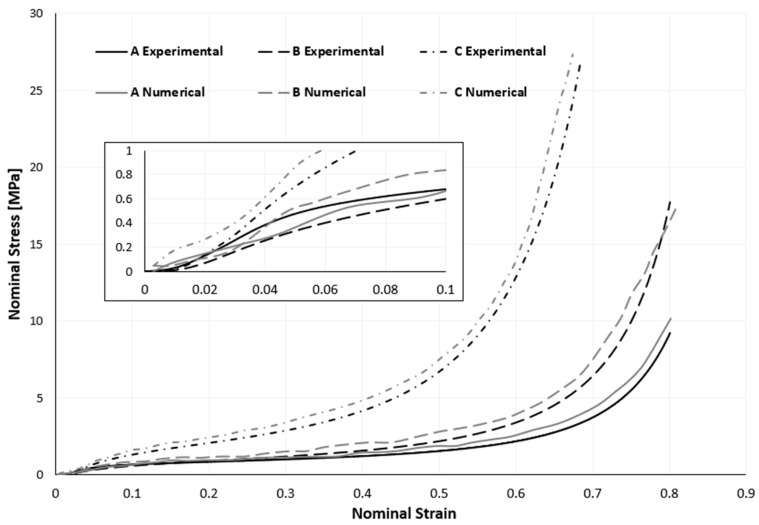
Variation between experimental and numerical stress–strain curves of quasi-static compression tests (the smaller graph is an enlarged section of the initial loading).

Although the numerical results exhibit stresses that are slightly higher than the experimental ones, it can be concluded from the numerical results and a comparison with the experimental results using Figure 8 that the numerical model accurately simulates the actual behaviour of the samples, with minimal deviation.

### 4.2. Dynamic Impact Tests

**Figure 9:**
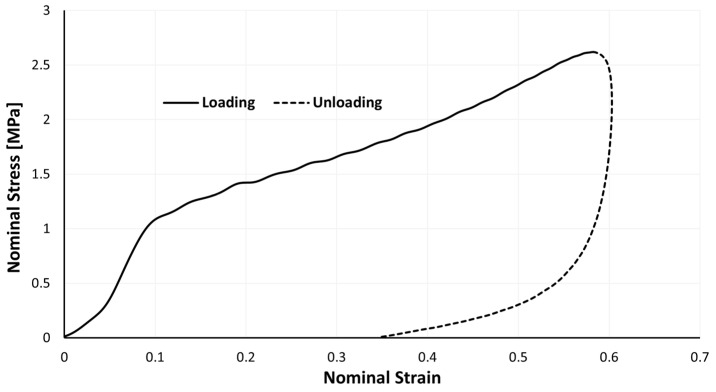
Loading and unloading (relaxation) phases of an impact test (type A sample).

It becomes clear through the examination of Figure 9 that, for dynamic impact tests, the stress–strain curve no longer resembles the traditional S-shaped curve of cellular materials under compression. The strain rate difference between the quasi-static and dynamic tests can explain this.

**Figure 10:**
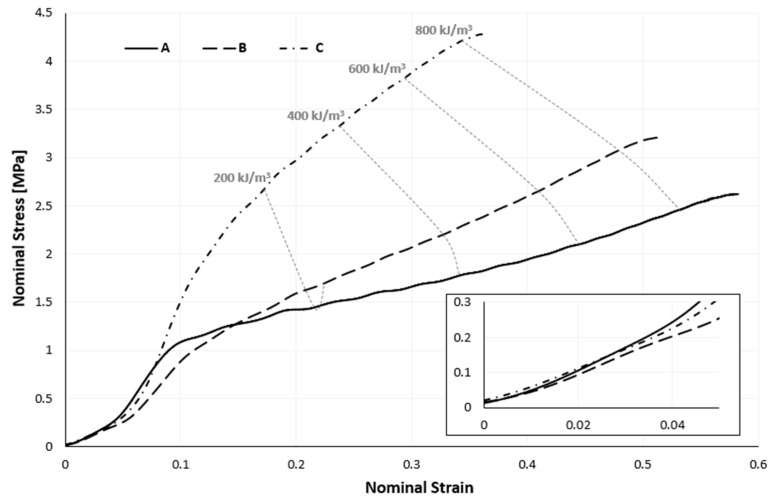
Nominal impact stress–strain curves of the three samples at four energy levels (the smaller graph is an enlarged section of the initial loading).

As a result, Figure 10 only emphasizes the portion of a stress–strain curve corresponding to the sample’s impact phase. The type A sample is the only one that makes it possible to distinguish between a linear elastic zone and a plateau region with varying degrees of ease. Up to 10% of the deformation in this sample can be regarded as the linear elastic zone and the remaining curve as the plateau zone, although the stress gradually rises in this zone. It is impossible to distinguish any of the three distinctive zones of the stress–strain curve for cellular materials under compression that are made of samples B or C.

By analyzing Figure 10, it is reasonable to conclude that the stress–strain curve tends to “open” as the sample density rises, reaching ever more significant stresses for more minor strains. A few mean parameters, along with their corresponding standard deviations (SDs), from the impact testing that support the previous conclusion are shown in Table 3.

Only the isocurve connected to the 200 kJ/m^3^ energy density has a distinctive, almost parabolic shape. All three isocurves, apart from the 200 kJ/m^3^ isocurve, have a negative slope; however, as the amount of absorbed energy increases, the slope tends to decrease.

However, as the samples’ densities increase, the samples’ energy densities decrease, as shown in Table 4. This is the reverse of what occurs with the stress–strain curves of the quasi-static compression experiments. Impact experiments with a 120 J impact energy were performed to verify this tendency. The energy density likewise declines with increasing sample density, as shown in Table 4. Although this discovery is intriguing, these energy levels are very comparable.

#### 4.2.1. Acceleration Peaks

One of the most important considerations when using cellular materials in safety devices is their maximum acceleration value, which is associated with the likelihood of injuries occurring. In order to prevent or limit injury to the user, it is ideal to obtain minimal acceleration peaks [24]. Similar to this, smaller acceleration peaks can protect the covered goods from more harm if the material is utilized in packaging.

**Figure 11:**
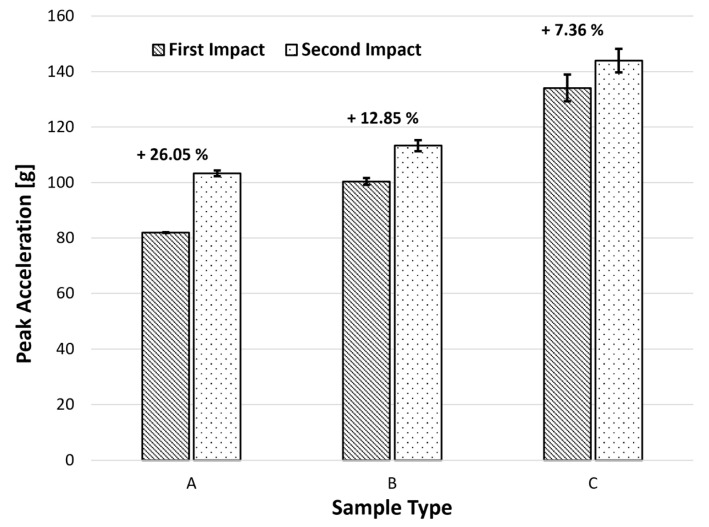
Influence of the sample’s density on its peak linear accelerations.

The peak linear acceleration (PLA) indeed relates to a deceleration, or, to put it another way, it relates to the impactor’s slowing acceleration. The same energy (60 J) was applied twice to three sets of samples with three different densities. The PLA of these impacts were recorded and are shown in Figure 11.

With a value of roughly 82 g, the sample with the lowest density (210 kg/m^3^) has the lowest peak acceleration during the first impact. Samples B and C, in contrast, attained accelerations of about 100 and 134 g, respectively. This acceleration increase with sample density can be explained by the fact that a lower density causes more cellular wall deformation and, as a result, a less abrupt acceleration. For the second impact, samples A, B, and C, respectively, recorded peak acceleration values of 103, 113, and 144 g. Thus, it is feasible to deduce that for samples A, B, and C, there was an increase in the acceleration between impacts of approximately 26, 13, and 7%, respectively. So, it follows that an increase in density will result in a smaller rise in acceleration between strikes. This can be explained by the fact that samples with a lower density are more likely to deform after their first impact, sustaining more damage and losing crashworthiness properties before the second impact.

#### 4.2.2. Rebound Velocity and Bounce-Back

**Figure 12:**
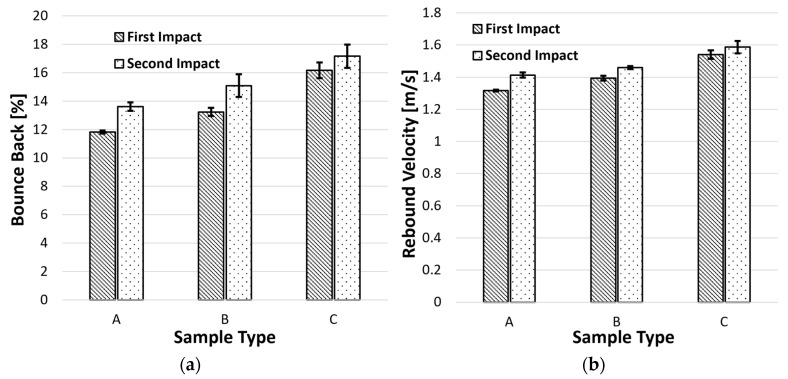
Influence of the density on (**a**) the bounce-back and (**b**) the rebound.

As a result of their viscoelastic properties, cork agglomerates can restore (almost completely) their original dimensions following deformation. The percentage of the impact energy immediately returned to the impactor is also measured and is now referred to as the bounce-back effect or rebound energy (Figure 12a). For safety devices, it is typically undesirable for a considerable portion of the impact energy to return to its load source. Therefore, a compromise between absorbed and returned energy must be maintained for devices enduring many impacts. Naturally, the bounce-back value increases from the first to the second impact for all samples examined. However, although cork grains retain their crashworthiness and elasticity after multiple collisions, the same cannot be said for polyurethane binders, which plastically absorb energy.

Furthermore, energy will be dispersed through microcracks formed in the cork grain/binder interfaces rather than being absorbed by plastic deformation during successive impacts. When the load is unloaded, more energy will be viscoelastically stored and released. The variance in bounce velocity between impacts, as shown in Figure 12b, further supports the claims above. The impactor’s exit velocity rises with increasing density. Additionally, all samples’ impactor output speeds increase from the first to the second impact.

#### 4.2.3. Unloading Phase

**Figure 13:**
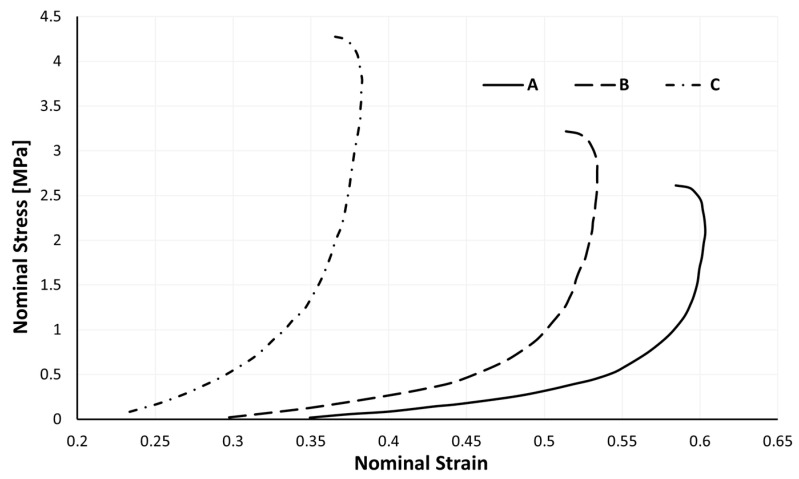
Stress–strain curve of the unloading phase of all three samples.

The relaxation phase, which is the period in which the impactor no longer presses on the sample, but rather the opposite, is going to be addressed. This crucial stage is nearly always overlooked. It is reasonable to infer from the analysis of Figure 13 that a higher sample density causes a shorter relaxation period for the samples’ dimensions. For samples A, B, and C, a strain recovery of around 23.5, 21.8, and 13.7%, respectively, is revealed. When conducting numerical simulations, and specifically when deciding the values of the material parameters *r*, *m*, and *β*, depicted in Section 2.1, it is crucial to keep in mind that the relaxation curve’s shape changes depending on the sample’s density.

#### 4.2.4. Impact Tests’ Numerical Results

The link between the stress and strain tensors is derived from the strain energy potential function *U*, which is unique to each hyperelastic model. The invariants (*I*_1_*, I*_2_*, I*_3_) of the strain tensor ***S***, which is determined by the deformation gradient tensor ***F***, are typically used to define the strain energy function *U*. The following relation is established using the deformation tensor ***B***, which is the left Cauchy–Green tensor:

(13) $$B=FF^{T}$$

The invariants of ***B*** are defined as follows:

(14) $$I_{1}=tr\left(B\right)=\lambda_{1}^{2}+\lambda_{2}^{2}+\lambda_{3}^{2}$$

(15) $$I_{2}=\frac{1}{2}\left[tr{\left(B\right)}^{2}-tr(B^{2})\right]=\lambda_{1}^{2}\lambda_{2}^{2}+\lambda_{2}^{2}\lambda_{3}^{2}+\lambda_{1}^{2}\lambda_{3}^{2}$$

(16) $$I_{3}=detB=J^{2}={\left(\det\left(F\right)\right)}^{2}=\lambda_{1}^{2}\lambda_{2}^{2}\lambda_{3}^{2}$$

where *λ_i_* are the principal stretches and *J* is the total volume ratio given by the determinant of the deformation gradient.

**Figure 14:**
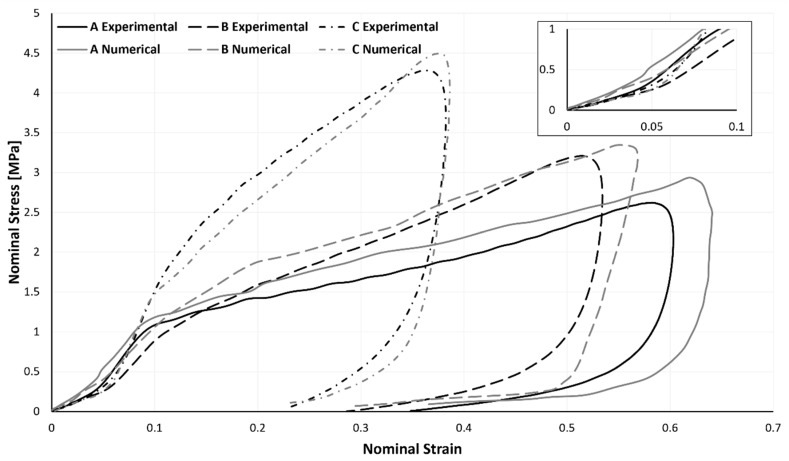
Variation between experimental and numerical stress–strain curves of the full impact test.

**Figure 15:**
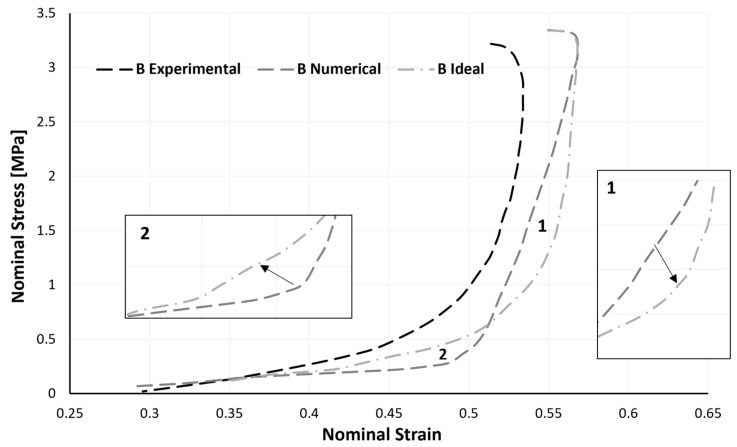
Difference in the experimental, numerical and ideal numerical results of the unloading phase of sample B.

Agglomerated cork was modelled as a nonlinear elastic solid. Due to the extremely low level of plasticity that was experimentally discovered, this simplification holds. Once again, agglomerated cork was modelled using a combination of the Hyperfoam and Mullins effect material models. The quasi-static compression curves shown in Figure 7, derived from a sample compressed at 5 mm/min, were scaled by a factor of 2.1, as mentioned earlier. To ascertain such a factor, independent dynamic compressive tests utilizing split Hopkinson pressure bars (SHPBs) or high-speed Zwick, MTC, Instron, or a comparable apparatus on cork samples are required for greater accuracy and scientific validity. However, because of the study facility’s technical constraints, a trial-and-error method involving multiple simulations determined this scaling factor value. Nevertheless, as seen in Figure 14 and Figure 15, this strategy worked and produced positive outcomes.

Additionally introduced into the model were the material densities, *ρ*, of 210, 300, and 400 kg/m^3^ for samples A, B, and C, respectively; the Poisson’s ratio, *ν*, which was approximately 0; and the strain energy potential order, *N*, which was of value 2. Table 5 presents these, as well as the other parameters added to the Mullins effect material model. A strain energy potential order of value 2 yielded more reliable results in this study.

The outcomes of the simulations and the experiments were contrasted. The entire (loading and unloading) stress–strain curve of both the numerical and experimental methods is shown in Figure 14. Similar to Table 3, Table 6 also contained the maximum force, acceleration, and strain values of the experimental and numerical data, allowing for their comparison and evaluation. Overall, as can be shown, the FEA findings are comparable to the ones measured experimentally, even if the numerical results show slightly greater stresses and strains.

It can be said that the values of the material parameters (r, *m,* and *β*) were correctly chosen because samples A and C exhibit relaxation behaviours that are remarkably comparable to those observed in the experiments during the period of recovery.

However, the numerical findings for the relaxation step in sample B may be enhanced. Along with the experimental and numerical data of sample B’s relaxation phase, Figure 15 also shows sample B’s ideal numerical behaviour. It would be better if the numerical curve shifted slightly to the right in the region denoted by number 1, which corresponds to the initial fall in stress. It would be ideal for the numerical behaviour not to display such a sharp apex in the region denoted by number 2, which is a region connected to the conclusion of the abrupt reduction in stress.

Recalling Figure 3, an attempt was made in zone 1 to raise the *m* parameter, and an attempt was made in zone 2 to lower the *β* parameter. Despite numerous attempts and parameter combinations, none proved to be numerically stable. So, in further work, this is an area that needs to be improved. Nevertheless, we can be said to achieve a numerical behaviour related to the relaxation phase that is close to the actual behaviour of the sample; the parameter *m* must likewise grow as the sample density does. Even if the model is not perfectly optimized and has a few flaws, when used in conjunction with critical thinking, it can be deemed to be acceptable and capable of providing accurate results.

**Figure 16:**
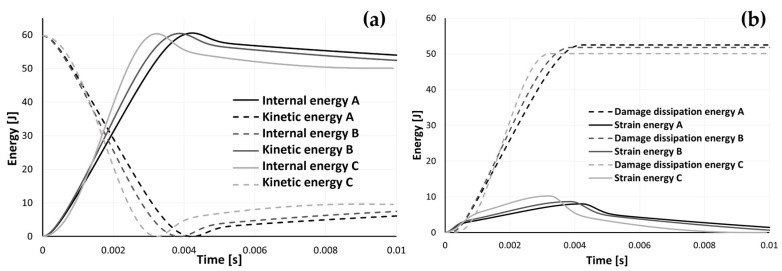
Time evolution of (**a**) internal and kinetic energy and (**b**) strain and damage dissipation energy.

Additionally, especially considering the substantial validation provided, Figure 16a,b display some numerical results. Both the model’s kinetic and internal energy are plotted in Figure 16a. In Figure 16b, the strain energy of the model and the amount of energy dissipated due to damage are displayed.

According to these findings, the model was able to dissipate 52.48, 51.77, and 50.09 J for samples A, B, and C, respectively, through damage. These values correspond to 87.8, 86.6, and 83.8% of the original kinetic energy, respectively. These numbers support the deductions made from the experimental findings that an increase in density causes a loss in the samples’ capacity to absorb energy during impact. Additionally, some energy is lost due to frictional effects, but since this amount is insignificant, we decided not to include it in these graphs. For samples A, B, and C, respectively, the kinetic energy reached its minimum at 4.2, 3.9, and 3.2 ms, indicating that the impactor’s velocity was zero and the rebound had just begun. As was previously observed, a rise in density encourages more abrupt deceleration. The maximum strain energy and damage dissipation energy occurred, as expected, at the same time as the minimum kinetic energy, which corresponds to the point of maximum deformation. Because the internal energy in this model is calculated as the sum of the strain energy and the damage dissipation energy, it also achieved its maximum level. Although the frictional energy’s dissipation was more negligible compared to the others in this model, it nonetheless had a role in the loss of impact energy.

Notably, the maximum internal energy values for models A, B, and C were 60.53, 60.44, and 60.36 J, respectively. The rule of the conservation of mechanical energy states that these numerical values of the internal energy are not physically feasible because they are higher than the initial gravitational potential energy and the kinetic energy at impact (59.78 J). These energy levels are excited, which may contribute to the explanation of why the numerical stress values are superior to the experimental stress results, as seen in Figure 14.

**Figure 17:**
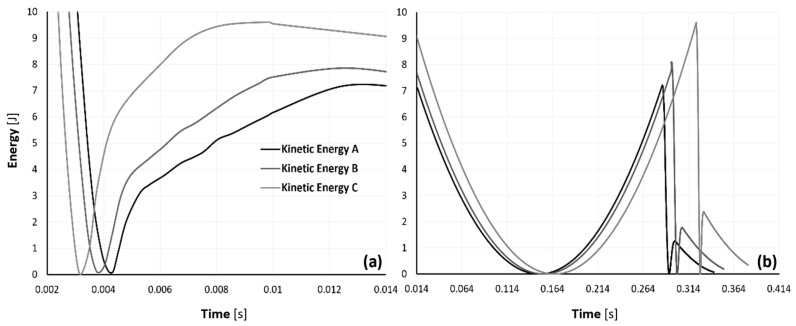
Time evolution of the kinetic energy of the model during the first two impacts: (**a**) initial impact and beginning of the ascent of the impactor, and (**b**) continuation of the ascent of the impactor, the descent of the impactor, and the second impact and second ascent of the impactor as it rebounds.

When the impactor’s kinetic energy was at its lowest, the rebound process began, and the impactor’s velocity rose until it lifted off of the sample. At this point, in Fernandes et al.’s [24] study, the velocity in the FEA remained constant, which meant that the kinetic energy stayed constant as well. However, since gravity was included in this model, we can see in Figure 17 that the kinetic energy begins to fall until it reaches zero, at which point it begins to increase again, simulating the rebound and subsequent beginning of the drop before the second hit.

**Figure 18:**
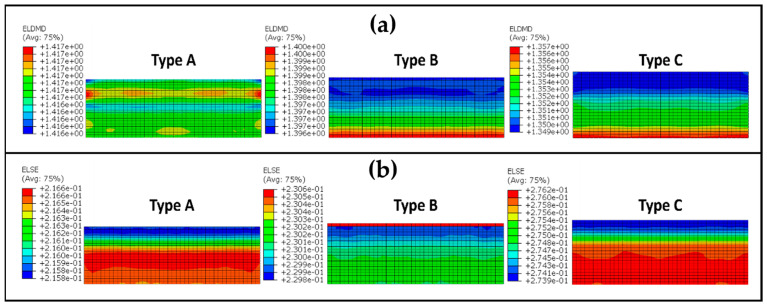
(**a**) Damage dissipation energy of the maximum deformation and (**b**) strain energy of the maximum deformation.

Finally, the region of the sample where the energy lost due to damage is greatest is its maximum deformation, which can be seen in Figure 18a. The damage dissipation energy curve in Figure 16b is produced by adding this variable to each node of the model. This process is also used to calculate the strain energy, and Figure 18b uses a similar depiction to 16a for the strain energy scenario. The ELDMD (total energy dissipated in the element by damage) and the ELSE (total elastic strain energy in the element) are represented in mJ.

## 5. Conclusions

Agglomerated cork offers a lot of promise for usage in situations where the ability to absorb energy is sought. Agglomerated cork was examined both statically and dynamically in this investigation. It should be noted that the stress–strain curve of these impact tests can and should be split into two sub-curves; one related to the impact and the other corresponding to the sample’s relaxation (Figure 9). These studies allowed the authors to draw the conclusion that agglomerated cork has a significant ability to rebound elastically, particularly at dynamic strain rates and when its permanent deformation was minimal (less than 2%). This enables the use of agglomerated cork in equipment that can be successfully reused and recycled after impact. This property makes this material ideal for energy absorption applications where many impacts may occur. This study advances our understanding of how to accurately simulate the mechanical behaviour of cork agglomerates under impact, and particularly the viscoelastic relaxation phenomenon, an aspect that researchers commonly ignore.

In our quasi-static studies, it was demonstrated that the energy absorption capacity increased proportionally with density. The type C sample, the densest, shows an increase of 38.6% in its energy absorption capacity compared to the type A sample, which is the least dense. However, the high rate of deformation, or the strain rate, in the dynamic impact testing seems to indicate that the samples’ ability to absorb energy decreases with increasing density and the influence of the significance of the Mullins effect. The type C sample shows a decrease of 4.6% and 2.6% in its energy absorption capacity compared to the type A and B samples, respectively. Given that the impact of agglomerated cork’s density on its mechanical behaviour under compression and impact was well established, this section of the work can be regarded as successful.

A numerical model for cork was further developed and optimized in this work. The constitutive model was examined against guided drop tests and quasi-static compressive tests. The actual behaviour of agglomerated cork during compression, as well as its relaxation after compression, could be accurately modelled using a mix of the Hyperfoam and Mullins effect material models. However, in the FEA, the Mullins effect was only applied to impacts, since there were no experimental data about the unloading phase of the quasi-static compressed tests. This can be viewed as a shortcoming of the work and ought to be fixed in later efforts to improve the numerical model’s precision and accuracy.

Despite being nonlinear elastic, the material model accurately captured the behaviour of agglomerated cork under dynamic compression (impacts). Additionally, the amount of permanent deformation observed in the studies was negligible. Since agglomerated cork compressions at dynamic strain rates can be simulated using this material model, the authors believe it to be valid. It was also possible to accurately and ultimately replicate the impactor’s impact, as well as its ascent and subsequent descent before the second impact, while taking into account the effects of gravity. This accomplishment can be viewed as an enhancement of a previous numerical model [24].

Finally, this work made it possible to successfully assess the impact of density on Mullins effect material parameters. It was possible to conclude that, for the numerical model to reproduce the actual relaxation behaviour of the sample faithfully, a rise in density must be accompanied by an increase in the parameter *m*. In contrast, the parameter β must stay approximately constant. Consequently, the constitutive approach of taking advantage of the built-in material models in Abaqus and tuning them can be deemed a real success. This study may and should be regarded as a significant contribution to the field of numerical mechanical modelling.

## Figures and Tables

**Table 1:** Design of the samples used in the experiments.

Material	Density [kg/m^3^]	Grain Size [mm]	Binder	Binder Content [wt.%]
A	210	2–4	PU Rigid	15
B	300	2–5	PU Rigid	14
C	400	2–4	PU Rigid	12

**Table 2:** Results of the absorbed energy density of the samples while under densification strain.

Material	Densification Strain	Energy Density [MJ/m^3^]
Type A	0.718	0.929
Type B	0.698	1.276
Type C	0.502	1.374

**Table 3:** Data on the sample impact tests that were performed.

Material	Density [kg/m^3^]	Peak Force [N]	SD Force	Peak Acceleration [g]	SD Acceleration	Peak Strain	SD Strain
Type A	210	6549.05	9.33	81.94	0.12	0.583	0.00346
Type B	300	8022.17	101.37	100.37	1.26	0.516	0.00531
Type C	400	10717.18	386.80	134.09	4.84	0.365	0.00575

**Table 4:** Results of the absorbed energy density of the samples and their densification strain under both 60 J and 120 J impact energies.

Impact Energy [J]	60		120	
Material	Strain	Energy Density [MJ/m^3^]	Strain	Energy Density [MJ/m^3^]
A	0.584	0.931	0.776	1.882
B	0.515	0.912	0.675	1.845
C	0.364	0.889	0.514	1.838

**Table 5:** Mullins effect parameters introduced to characterize agglomerated cork.

Material	Density [kg/m^3^]	Poisson Ratio	*N*	*r*	*m*	β
Type A	210	≈0	2	1.1	0.07	0.01
Type B	300				0.08	0.02
Type C	400				0.14	0.01

**Table 6:** Comparison of the data from sample impact tests that were experimentally performed and numerically simulated.

Material	Peak Force [N]		Peak Acceleration [g]		Peak Strain	
	Exp.	Num.	Exp.	Num.	Exp.	Num.
Type A	6459.05	6789.98	81.94	86.13	0.364	0.365
Type B	8022.17	8363.25	101.37	105.68	0.521	0.551
Type C	10,717.18	11,813.88	134.09	147.81	0.591	0.627

## Data Availability

The original contributions presented in the study are included in the article, further inquiries can be directed to the corresponding author.

## References

[1] Alcântara I., Teixeira-Dias F., Paulino M. (2013). Cork composites for the absorption of impact energy. Compos. Struct..

[2] Paulino M., Teixeira-Dias F. (2011). An energy absorption performance index for cellular materials—Development of a side-impact cork padding. Int. J. Crashworthiness.

[3] Gibson L., Ashby M. (1997). Cellular Solids: Structure and Properties.

[4] Fernandes F.A.O., Alves de Sousa R.J. (2013). Motorcycle helmets—A state of the art review. Accid. Anal. Prev..

[5] Landro D.L., Sala G., Olivieri D. (2002). Deformation mechanisms and energy absorption of polystyrene foams for protective helmets. Polym. Test..

[6] Mills N.J., Stämpfli R., Marone F., Brühwiler P.A. (2009). Finite elements micromechanics model of impact compression of closed-cell polymer foams. Int. J. Solids Struct..

[7] Ouellet S., Cronin D., Worswick M. (2006). Compressive response of polymerics foams under quasi-static, medium and high strain rate conditions. Polym. Test..

[8] Fernandes F.A.O., Jardin R.T., Pereira A.B., Alves de Sousa R.J. (2015). Comparing the mechanical performance of synthetic and natural cellular materials. Mater. Des..

[9] Ptak M., Kaczynski P., Fernandes F.A.O., Alves de Sousa R.J. (2017). Assessing impact velocity and temperature effects on crashworthiness properties of cork material. Int. J. Impact Eng..

[10] Kaczynsky P., Ptak M., Wilhelm J., Fernandes F.A.O., Alves de Sousa R.J. (2019). High-energy impact testing of agglomerated cork at extremely low and high temperatures. Int. J. Impact Eng..

[11] Pereira H. (2007). Cork: Biology, Production and Uses.

[12] Gil L. (2017). Patent search on cork (2010–2015). Ciência Tecnol. Mater..

[13] Castro O., Silva J.M., Devezas T., Silva A., Gil L. (2010). Cork agglomerates as an ideal core material in lightweight structures. Mater. Des..

[14] Suffo M., Sales D.L., Cortés-Triviño, de la Mata M., Jiménez E. (2022). Characterization and production of agglomerated cork stoppers for spirits based on a factor analysis method. Food Packag. Shelf Lide.

[15] Serra G., Fernandes F.A.O., Alves de Sousa R.J., Noronha E., Ptak M. (2022). New hybrid cork-STF (shear thickening fluid) polymeric composites to enhance head safety in micro-mobility accidents. Compos. Struct..

[16] Buil R.M., Angulo D.R., Ivens J., Blasco J.O.A. (2021). Experimental study of natural cork and cork agglomerates as a substitute for expanded polystyrene foams under compressive loads. Wood Sci. Technol..

[17] Antunes e Sousa G.J., Rocha A., Serra G., Fernandes F.A.O., Alves de Sousa R.J. (2023). Shear thickening fluids in cork agglomerates: An exploration of advantages and drawbacks. Sustainability.

[18] Fortes M.A., Nogueira M.T. (1989). The poison effect in cork. Mater. Sci. Eng. A.

[19] Anjos O., Pereira H., Rosa M.E. (2008). Effect of quality, porosity and density on the compression properties of cork. Eur. J. Wood Wood Prod..

[20] Sanchez-Saez S., Barbero E., Garcia-Castillo S.K., Ivañez I., Cirne J. (2015). Experimental response of agglomerated cork under multi-impact loads. Mater. Lett..

[21] Anjos O., Rodrigues C., Morais J., Pereira H. (2014). Effect of density on the compression behaviour of cork. Mater. Des..

[22] Santos P.T., Marques P.A.A.P., Pereira A.B., Alves de Sousa R.J. (2017). Agglomerated cork: A way to tailor its mechanical properties. Compos. Struct..

[23] Gameiro C.P., Cirne J., Gary G., Miranda V., Pinho-da-Cruz J., Teixeira-Dias F. (2006). Numerical and experimental study of the dynamic behaviour of cork. 3rd Light-Weight Armour Group Workshop: Design and Use of Light-Weight Materials.

[24] Fernandes F.A.O., Pascoal R.J.S., Alves de Sousa R.J. (2014). Modelling impact response of agglomerated cork. Mater. Des..

[25] Gomez A., Barbero H., Sanchez-Saez S. (2022). Modelling of carbon/epoxy sandwich panels with agglomerated cork core subjected to impact loads. Int. J. Impact Eng..

[26] Johnson A.F. (2001). Modelling fabric reinforced composites under impact loads. Compos. Part A Appl. Sci. Manuf..

[27] Ladeveze P., Ledantec E. (1992). Damage modelling of the elementary ply for laminated composites. Compos. Sci. Technol..

[28] Tsai S.W., Wu E.M. (1971). A General Theory of Strength for Anisotropic Materials. J. Compos. Mater..

[29] Sergi C., Boria S., Sarasini F., Russo P., Vitiello L., Barbero E., Sanchez-Saez S., Tirillò J. (2021). Experimental and finite element analysis of the impact response of agglomerated cork and its intraply hybrid flax/basalt sandwich structures. Compos. Struct..

[30] Sergi C., Sarasini F., Russo P., Vitiello L., Barbero E., Sanchez-Saez S., Tirillò J. (2022). Experimental and numerical analysis of the ballistic response of agglomerated cork and its bio-based sandwich structures. Eng. Fail. Anal..

[31] (2010). ABAQUS 6.10 Documentation.

[32] Hacket R.M. (2016). Strain-Energy Function. Hyperelasticity Primer.

[33] Smith M. (2009). ABAQUS/Standard User’s Manual, Version 6.9.

[34] PolymerFEM (2021). Parametric Study of the Mullins Effect Model. https://polymerfem.com/parametric-study-of-the-mullins-effect-model/.

